# Regulation of Store-Operated Ca^2+^ Entry by SARAF

**DOI:** 10.3390/cells10081887

**Published:** 2021-07-26

**Authors:** Inbal Dagan, Raz Palty

**Affiliations:** Technion Integrated Cancer Center, Department of Biochemistry, Ruth and Bruce Rappaport Faculty of Medicine, Technion—Israel Institute of Technology, Haifa 31096, Israel; inbal.dagan@campus.technion.ac.il

**Keywords:** store operated calcium entry (SOCE), STIM1, SARAF, Orai1, CRAC channel, slow calcium dependent inactivation (CDI)

## Abstract

Calcium (Ca^2+^) signaling plays a dichotomous role in cellular biology, controlling cell survival and proliferation on the one hand and cellular toxicity and cell death on the other. Store-operated Ca^2+^ entry (SOCE) by CRAC channels represents a major pathway for Ca^2+^ entry in non-excitable cells. The CRAC channel has two key components, the endoplasmic reticulum Ca^2+^ sensor stromal interaction molecule (STIM) and the plasma-membrane Ca^2+^ channel Orai. Physical coupling between STIM and Orai opens the CRAC channel and the resulting Ca^2+^ flux is regulated by a negative feedback mechanism of slow Ca^2+^ dependent inactivation (SCDI). The identification of the SOCE-associated regulatory factor (SARAF) and investigations of its role in SCDI have led to new functional and molecular insights into how SOCE is controlled. In this review, we provide an overview of the functional and molecular mechanisms underlying SCDI and discuss how the interaction between SARAF, STIM1, and Orai1 shapes Ca^2+^ signaling in cells.

## 1. Introduction

Store-operated calcium entry (SOCE) is an evolutionarily conserved mechanism present in nearly all types of metazoan cells. SOCE operates to replace Ca^2+^ lost from the endoplasmic reticulum (ER) with Ca^2+^ that enters the cytoplasm through plasma membrane (PM) channels [1]. The transmembrane Ca^2+^ flux replenishes luminal ER Ca^2+^ and plays a critical role in many cellular functions, including secretion, migration, and gene expression. Accordingly, numerous studies have shown that abnormalities in SOCE are associated with a variety of disease states, including cancer, neurodegeneration, and cardiovascular diseases. Moreover, gain or loss of function mutations in genes of the key mediators of SOCE lead to multi-systemic disorders, including immunodeficiency and autoimmunity, muscle hypotonia and skeletal muscle myopathy, ectodermal dysplasia, and mydriasis [2,3]. Though several members of TRPC family of ion channels contribute to SOCE [4], the calcium release-activated calcium (CRAC) channel is the prototypical mediator of this process in most types of cells [3]. The molecular identity of the channel remained a mystery for decades and was eventually solved in 2005–2006 with the identification of two key components, stromal interaction molecule (STIM) and Orai [5,6,7,8,9,10,11]. STIM is the ER Ca^2+^ sensor that detects changes in ER Ca^2+^ levels and responds by coupling to Orai, the Ca^2+^ channel located in the PM, to open the channel and initiate cellular Ca^2+^ entry. Mammalian cells contain two STIM isoforms, STIM1 and STIM2, and three Orai isoforms (ORAI1–3); however, much of the current knowledge of the structure-function of CRAC channels and their physiological roles arises from studies involving STIM1 and Orai1 [12]. Seminal structural work by Long and colleagues revealed that channels of the fly orthologue of mammalian Orai1 are arranged as hexamers [13]. Each Orai subunit has four transmembrane segments with residues from TM1 lining the channel pore while residues from TM2–4 organize in concentering rings around it. Both N- and C-termini of each subunit face towards the cytosol, and pairs of neighboring C-termini bend towards each other and interact when the channel is closed and disengage when the channel opens [14,15]. The C-terminus of Orai1 contains a key site that is necessary and sufficient for STIM1 binding [15,16]; however, both N- and C-termini cooperate in binding to STIM1 and in conveying the gating signal to the pore [17,18,19,20,21,22]. STIM1 is a single pass membrane protein that localizes to endoplasmic reticulum membrane. The luminal-facing EF-SAM domain of STIM1 harbors multiple calcium-binding sites that sense changes in luminal calcium levels [23,24,25]. Unbinding of Ca^2+^ from this domain of STIM1 induces rearrangement of the transmembrane domain, which in turn elicits a large conformational change in the cytosolic-facing part of the protein [26,27,28,29,30,31,32,33,34]. The cytosolic-facing part of STIM1 contains multiple functional and regulatory domains, including the STIM1-Orai1 activation region (SOAR [35], also referred to as CAD [36]), a short segment that is both necessary and sufficient for binding and gating the Orai1 channel. Under resting conditions, the SOAR is shielded by intramolecular inhibitory mechanisms that prevent it from spontaneously binding to Orai1. The first of these involves a well-characterized interaction between the SOAR and the CC1 (coiled coil 1) region that is located between TM and SOAR [27,32,34]. The second involves a recently described interaction between the STIM1 inhibitory domain (STIM1 ID), which is located C-terminally to SOAR, with the membrane proximal segment of the CC1 region [37,38]. Depletion of ER Ca^2+^ induces rearrangement of the STIM1 TM region, which in turn leads to pairing of neighboring CC1 regions [26,27,28,29,30,32]. Ligation of CC1 regions removes both brakes from SOAR and stimulates multimerization of STIM1. These conformational changes also liberate a lipid-binding region at the C-terminus, allowing STIM1 multimers to interact with plasma membrane phospholipids and accumulate at ER-PM contact sites [6,23,39,40,41]. At these active sites, STIM1 binds to and gates Orai1 channels to elicit Ca^2+^ influx into cells.

Under physiological conditions, the rise in intracellular Ca^2+^ stimulates various intracellular calcium-dependent signaling cascades. However, if left unchecked, prolonged elevation in cytosolic Ca^2+^ may also induce cytotoxicity and cell death [42]. Consequently, a number of inactivation mechanisms have evolved to constrain channel activity. Key among these is a process called calcium-dependent inactivation (CDI), which represents a feedback mechanism that operates to balance the excess of cytosolic calcium in cells. Inhibition of CRAC channel function by calcium takes place in two basic ways, channel deactivation and channel inactivation. Channel deactivation occurs when Ca^2+^ levels rise both in the cytosol and the ER lumen and involves both physical and functional decoupling between STIM1 and Orai1, accompanied by a reorganization of both proteins back to their resting conformations [23,33,43,44,45,46]. Conversely, CDI only requires a rise in cytosolic calcium and does not appear to involve a comprehensive physical disassembly of the channel complex. CDI occurs through two temporally distinct mechanisms of fast (FCDI) and slow (SCDI) inactivation. FCDI takes place on a millisecond time frame (with τ1/2 of ~10 and 100 ms) and involves regulatory regions located on both STIM1 and Orai1 [47,48,49,50,51]. On the other hand, SCDI develops gradually over tens of seconds and depends on multiple mechanisms that are both intrinsic and extrinsic to the CRAC channel [37,52,53,54,55,56,57]. One of the best characterized regulators of SCDI in cells is the SOCE-associated regulatory factor SARAF. In this contribution, we begin with a mechanistic overview of CRAC channel SCDI and focus on the idea that SCDI emerges from a number of distinct mechanisms in cells. We then review the current state of the field for studies on the molecular basis for CRAC channel regulation by SARAF. Finally, we discuss the physiological roles of SARAF in light of recent studies from SARAF KO mice.

## 2. Slow Calcium-Dependent Inactivation—What Is the Mechanism?

A defining feature of CRAC channel SCDI in cells is the inhibition of channel activity that develops over tens of seconds and depends on a rise in cytoplasmic Ca^2+^ [53,54]. Although first characterized more than two decades ago, the mechanism underlying SCDI remains poorly understood. A major contributing factor to this vagueness is the apparent heterogeneous behavior of SCDI in different types of cells. Initial indication of the phenomenon was reported by Louzao et al., showing that the effects of BAPTA and EGTA, Ca^2+^ chelators with similar Ca^2+^ affinity but different binding kinetics, on slow inactivation are similar in lacrimal and acinar cells but different in fibroblasts [58]. Most studies, however, employed either rat basophilic leukemia (RBL), Jurkat T-lymphocytes, or human embryonic kidney cells (HEK293 cells) to investigate SCDI of CRAC channels. Comparison of functional characteristics of SCDI in these cells further suggests that different mechanisms are at play in each cell type (summarized in Table 1).

In the three types of cells, dialysis with high levels of BAPTA (~8–10 mM) fully suppressed SCDI whereas moderate amounts of the slow Ca^2+^ chelator EGTA (1.2–1.4 mM) led to about ~50% inhibition [52,53,54,61]. However, while dialysis with high levels of EGTA (10–14 mM) fully blocked SCDI in RBL cells [54,60], it produced diverse effects in Jurkat cells [53,59] and did not prevent inactivation in HEK293 cells [57,61]. These differences suggest that in each type of cell the mechanism that triggers SCDI is located at a different distance from the channel pore [64]. In RBL cells, the similar sensitivity to high concentrations of either EGTA or BAPTA places the Ca^2+^ nanodomain of SCDI at a distance larger than 100 nm from the CRAC channel pore, while in Jurkat T-lymphocytes [59] or HEK293 [57,61], where SCDI is effectively blocked by high levels of BAPTA but not by similar levels of EGTA, the Ca^2+^ nanodomain is likely to be much closer to the channel pore (within 10–50 nm). Further signifying that different mechanisms mediate SCDI in these cells, SCDI shows sensitivity to Ca^2+^ with half maximal inhibition at around ~0.5 μM in RBL cells [62] and ~0.2 μM in HEK293 cells [61]. Furthermore, SCDI does not require a continuous presence of elevated Ca^2+^ in RBL cells [54], while in HEK293 [52,61] and Jurkat [53] cells, following a transient decrease in Ca^2+^ levels, the current recovers partially (HEK293 cells) or fully (Jurkat cells). The basis for these differences is presently unknown but may involve changes in the subunit composition of CRAC channels [61], expression of regulators of STIM1 or Orai1 [52,55,56], or other mechanisms that contribute to localization of CRAC channels at ER-PM contact sites [63], or to local elevation [65] or buffering [59,60] of Ca^2+^ near CRAC channels.

Indeed, Lis et al. showed that the different isoforms of Orai exhibit distinct functional properties [61]. Whereas currents by Orai1 channels exhibit sensitivity to SCDI, those by Orai2 and Orai3 do not [61]. It is also worth mentioning that although it is not presently known if the sensitivity of the Orai channel to SCDI is lost or retained when Orai1 multimerizes with Orai2 or Orai3, different heteromeric assemblies of Orai in different cell types are also likely to contribute to differences in CRAC channel SCDI.

The different IC50 profiles of SCDI in HEK293 and RBL cells [61,62] may arise from two separate sensing mechanisms with different Ca^2+^ affinities. Calmodulin (CaM) was reported as a putative Ca^2+^ sensor for SCDI in HEK293 cells [56]. Consistent with the notion that SCDI is mediated by distinct Ca^2+^ sensing mechanisms in HEK293 and RBL cells, pharmacological inhibition of CaM suppresses SCDI in HEK293 [56] but not in RBL cells [54]. The cell-type specific effect of CaM inhibition is somewhat surprising since CaM is ubiquitously expressed and highly abundant in cells. However, this inconsistency may be explained by the different ways through which CaM has been shown to affect the CRAC channel, including the binding of CaM to Orai1 [66,67] or to the SOAR of STIM1 [56], which promotes the decoupling of STIM1 from Orai1, and the interaction of CaM with the lipid-binding C-terminus of STIM1 or STIM2 [68,69], which may interfere with the targeting of STIM1 to ER-PM junctions and therefore with SCDI [63].

Calcium buffering by mitochondria has also been shown to modulate SCDI, suggesting that mitochondrial heterogeneity is another important source for cell-type dependent differences in SCDI. The steep membrane potential (~−180 mV) across the mitochondrial inner membrane that drives ATP synthesis also enables robust and rapid calcium uptake into the mitochondrial matrix. Gilabert and Parekh reported strong SCDI when intracellular Ca^2+^ was buffered by 0.1 mM EGTA but almost no inactivation with 10 mM EGTA in RBL cells [60]. Under low intracellular Ca^2+^ buffering (0.1 mM EGTA), inclusion of a cocktail of intermediary metabolites required for mitochondrial respiration in the patch pipette strongly suppressed SCDI and this effect was reversed by ruthenium red, a blocker of mitochondrial Ca^2+^ uptake [60]. A subsequent study by Gilabert and collaborators analyzed SCDI in Jurkat T-cells and showed that the release of ATP by mitochondria localized close to CRAC channels increases Ca^2+^ buffering capacity and suppresses SCDI [59]. Hence, different cell-dependent mechanisms by mitochondria contribute to buffering of free cytosolic Ca^2+^ and hence to suppression of SCDI. A search for regulators of mitochondrial Ca^2+^ led to a serendipitous identification of SARAF, a key regulator of SCDI [52]. As elaborated in the following sections, the contribution of SARAF to the regulation of SOCE also exhibits cell-type dependent variations. Therefore, while the molecular basis of SCDI is presently unclear, the idea that it arises from distinct mechanisms operating in a cell-type specific manner should be instrumental to the design and execution of future studies of this inhibitory pathway.

## 3. Molecular Identification of SARAF

SARAF was identified by a high throughput functional screen for modulators of mitochondrial matrix calcium. Although SARAF localizes primarily to the ER membrane [52], and also partially to the PM [70] but not mitochondria, its overexpression caused a significant decrease in resting mitochondrial calcium. This conundrum was resolved by analysis of changes in the levels of cytosolic and ER Ca^2+^ which demonstrated that increased expression of SARAF caused a global change in cellular Ca^2+^ homoeostasis by modulating Ca^2+^ fluxes across the plasma membrane. A combination of Ca^2+^ imaging and electrophysiological analyses further revealed that the pathway sensitive to the effect of SARAF is SOCE and that SARAF facilitates CRAC channel SCDI in both HEK293 and Jurkat T-cells [52]. Although subsequent studies found that SARAF also affects the function of arachidonic acid-sensitive channels [71] and TRPC1 channels [72], the main target of SARAF appears to be CRAC channels. Confirming a key role for SARAF in both CRAC channel deactivation and SCDI, a number of reports showed that while an increase in SARAF expression promotes channel inhibition, a reduction therein causes the opposite effect [37,63,73,74]. Investigations of the mechanism behind CRAC channel regulation by SARAF yielded a considerably more complex picture.

## 4. Interaction of SARAF with the CRAC Channel

**Figure 1:**
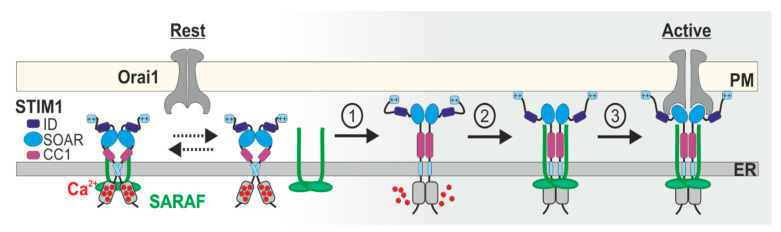
Interaction between the CRAC channel and SARAF during channel activation. Rectangle and oval symbols indicate the different domains of STIM1. At resting conditions, a fraction of SARAF (green) interacts with STIM1. Following ER Ca^2+^ depletion (step #1), SARAF undergoes a transient disassociation from STIM1 but the interaction is quickly regained (step #2) and further augmented (step #3) following binding of STIM1 to Orai1.

Physical interaction between SARAF and STIM1 is well documented, and the dynamics of this interaction are summarized in Figure 1. Upon heterologous expression of fluorescent protein-tagged versions of SARAF and STIM1 in cells, FRET measurements place the two proteins within a 2–10 nm distance and protein pull-down analyses indicate that they occupy the same protein complex [37,52,63]. Protein co-immunoprecipitation (coIP) studies revealed that when incubated with lysates prepared from cells expressing STIM1 and Orai1, a purified fragment of the C-terminal region of SARAF mainly precipitated STIM1 proteins, indicating that STIM1 is the key binding partner of SARAF [52]. The TM and ER luminal-facing domains of SARAF were not required for this interaction, placing the site of STIM1-SARAF interaction in the cytosol [52]. Consistent with this conclusion, additional coIP studies showed that the SOAR is the key binding site for SARAF in STIM1 [37] and that physical interaction between full length STIM1 and SARAF occurs at resting conditions [37,63,73,75,76], transiently decreases during the first minute after ER Ca^2+^ depletion but is regained shortly afterwards (1–2 min) [63,75,76], and exhibits sensitivity to mutations in the STIM1 CC1 [73] and CTID [37], which control the accessibility of SOAR. Total internal reflection microscopy (TIRFM) and Forester resonance energy transfer (FRET) studies added important insights related to the cellular localization and dynamics of this interaction. The analyses showed that following ER Ca^2+^ depletion, SARAF translocates to ER-PM contact sites in a STIM1-dependent manner, that this step is accompanied by augmentation of STIM1-SARAF interaction after STIM1 binds to Orai1 [52,63], and that it occurs specifically at membrane microdomains enriched with phosphatidylinositol 4,5-bisphosphate (PI(4,5)P2) and stabilized by E-Syt1 and septin 4 [63]. The interaction is reversible and returns to basal levels after ER Ca^2+^ refilling [52]. An interesting and highly important point is that almost all supporting data for the dynamic increase in interaction between SARAF and the STIM1-Orai1 channel complex were obtained under conditions that do not elicit SCDI. Therefore, SARAF already physically engages the CRAC channel during channel activation and prior to induction of SCDI. While the functional significance of this early interaction is presently unclear, it may indicate an initial role for SARAF in channel activation [76,77] and raises a critical question regarding how the binding of SARAF to STIM1 ultimately promotes SCDI.

## 5. Current Models for SCDI by SARAF

Two related and presently unresolved questions are (1) how does the interaction between SARAF and the CRAC channel complex promote channel inhibition? and (2) what is the molecular basis for the Ca^2+^ sensitivity of CRAC channel inhibition by SARAF?

By expressing a series of STIM1 deletion mutants in HEK293 cells, Jha et al. examined the contribution of different regions in the C-terminus of STIM1 to CRAC channel function and to the interaction with SARAF [37]. The study identified a region in STIM1 called the C-terminal Inhibitory Domain (CTID, amino acids 448–532), which is located C-terminally to SOAR and operates both to keep the SOAR shielded from interaction with Orai1 under resting conditions and to regulate its interaction with SARAF [37]. Subsequent work by Lee et al. narrowed the critical inhibitory region within the CTID to residues 470–491, which encompass the STIM1 inactivation domain (STIM1 ID), and showed that this site interacts with the CC1 region to maintain STIM1 in its resting conformation [38]. Importantly, the CTID study showed that SARAF binds to SOAR and partially inhibits Orai1 currents induced by SOAR when Ca^2+^ is buffered by low levels of EGTA (1.2 mM) but not when it is buffered by higher levels (10 mM) of BAPTA [37]. This finding indicates that SARAF inhibits SOAR only when Ca^2+^ levels are elevated and, since inhibition was about ~50% of that observed for full length STIM1, other regions (i.e., the CTID) in STIM1 are required for the full inhibitory effect [37]. The study further showed that while SARAF does not bind directly to the CTID, this regulatory site controls the interaction between SARAF and SOAR. Deletion of a section of the CTID located proximally to SOAR (amino acids 448–490) enhanced both the interaction between STIM1 and SARAF as well as CRAC channel inhibition, while interference with the section of the CTID that is more distal to SOAR (amino acids 490–532) caused opposite effects. Based on these findings, a model was presented whereby the two lobes of the CTID play differential roles in the inhibition of CRAC channel function by allowing or restricting the access of SARAF to SOAR. In the resting state, the distal lobe directs SARAF to SOAR to maintain the resting conformation of STIM1. Upon STIM1 activation, the proximal lobe transiently restricts access of SARAF to SOAR and allows SOAR to interact with and activate Orai1. Finally, an additional conformational transition in the STIM1 CTID, presumably triggered by a rise in cytosolic Ca^2+^, supports the re-interaction of SARAF with SOAR to promote channel inhibition [37].

The CTID model provides a useful framework that accounts for a number of observations related to the dynamics of STIM1-SARAF interaction and the dual inhibitory effect of SARAF at both rest and activated states of STIM1. However, the model is inconsistent with several observations from other reports. For example, analysis of FRET between STIM1 and SARAF before and after ER Ca^2+^ depletion shows only an increase in interaction and not the transient bidirectional interaction change proposed by the model [52,63]. As discussed throughout the current work, there are multiple lines of evidence for non-inhibitory interaction between SARAF and STIM1 after ER Ca^2+^ depletion and prior to induction of SCDI, which is not accounted for by the model. It is also not clear that the CTID is indeed essential for maintaining the resting conformation of STIM1 since a STIM1 deletion construct truncated at residue 448 (STIM1 1-448), and hence lacking the entire CTID, exhibits normal store-operated coupling to Orai1 [36,78]. This indicates that spontaneous activation of Orai1 by the STIM1 ID or CTID deletion mutants depends on the C-terminal region after the CTID (residues 530–685). The corresponding C-terminal region of STIM1 contains sites important for microtubule tracking [79,80] and for stabilizing interaction of STIM1 with PM phospholipids [81,82,83,84,85]. Intriguingly, both functions have been shown to regulate Orai1 channel inhibition. Enhancement of STIM1 binding to EB1 has recently been shown to delay translocation of STIM1 to ER-PM junctions and consequently prevents excess of SOCE [86], and the lysine-rich C-terminus of STIM1 was shown to be critical for SCDI and for interaction with SARAF in HEK293 cells [63]. Hence, the CTID model seems to provide only a partial account of the contribution of SARAF to regulation of CRAC channels.

A report by Albarran et al. added additional complexity to the CTID model and provided evidence that prior to promoting CRAC channel inhibition, SARAF initially contributes to channel activation [76]. The study showed that shortly after STIM1 activation by ER Ca^2+^ depletion, SARAF dissociates from STIM1 and binds transiently to the C-terminus of Orai1 [76]. Ca^2+^ imaging experiments in murine hybridoma cancer cells demonstrated that purinergic stimulation was followed by enhancement of cellular Ca^2+^ entry. This PM Ca^2+^ flux was sensitive to changes in expression of either Orai1 or SARAF, suggesting that binding of SARAF to Orai1 stimulated channel activation [76]. In line with a stimulatory role for SARAF during SOCE activation, Ca^2+^ imaging experiments in human endothelial cells showed that RNAi-mediated knockdown of Orai1 or SARAF expression attenuates VEGF-induced Ca^2+^ entry [77]. Because Albarran et al. had used cells with low expression levels of STIM1, their model suggested that SARAF contributes to Orai1 channel activation independently of STIM1 [76]; however, it is not clear that the contribution of SARAF to Ca^2+^ entry in these cells was indeed STIM-independent as it may have involved STIM2, which also mediates SOCE in cells [12] and which is also regulated by SARAF [52]. Further uncertainty for a STIM-independent effect of SARAF on Orai1 function arises from studies in HEK293 cells which showed that SARAF fails to reach ER-PM sites in the absence of STIM1 [52] and that the deletion of both STIM1 and STIM2 is sufficient to fully block Ca^2+^ entry [87] despite significant levels of endogenous SARAF and Orai1 proteins in these cells [52,63]. Finally, reduced levels of either Orai1, SARAF, or STIM1 attenuate Ca^2+^ entry in endothelial cells, suggesting that all three components affect SOCE in these cells [88,89]. The bi-directional effect of SARAF on CRAC channel activation and inactivation is highly intriguing and suggests a broader role for SARAF in the regulation of SOCE. Clearly, additional work is necessary to elucidate exactly how SARAF participates in either mechanism of CRAC channel function.

## 6. The Molecular Basis for Ca^2+^ Sensitivity of SCDI by SARAF

In both of the above mentioned models, binding of SARAF to the SOAR of STIM1 under high levels of cytosolic Ca^2+^ promotes inhibition of CRAC channel function [37,63,76]. The models, however, remain ambiguous as to whether SARAF physically interacts with SOAR in a Ca^2+^-independent manner and promotes inactivation only when cytosolic Ca^2+^ levels rise, or alternatively, whether a rise in cytosolic Ca^2+^ elicits an interaction between SARAF and SOAR that inhibits its ability to activate Orai1. Currently, based on a large body of evidence from multiple studies, the first possibility appears more likely. Demonstrating a type of SARAF-STIM1 interaction that does not require Ca^2+^, co-IP analyses showed that SARAF binds to either full length STIM1 [37,52,63,75,76] or SOAR alone [37] under conditions in which the levels of free Ca^2+^ in the cytosol are expected to be low (i.e., resting levels of about ~50–100 nM). FRET experiments also show a significant interaction between STIM1 and SARAF under resting conditions [52,63]. Both coIP and FRET studies, however, show an increase in SARAF–STIM1 interaction after depletion of ER Ca^2+^ with SERCA-inhibiting compounds [52,63,73,75,76]. This change might represent a form of Ca^2+^-dependent interaction since application of SERCA inhibitors causes a transient rise in cytosolic Ca^2+^. However, this scenario appears doubtful because the increase in STIM1-SARAF FRET is not transient, as would be expected from a Ca^2+^-dependent process [52,63] and is unaffected by elevation of intracellular Ca^2+^ following induction of SOCE [52], and since coIP studies indicate that complexes of SARAF and STIM1 obtained under resting or ER Ca^2+^-depleted conditions are not sensitive to prolonged incubation (~4–8 h) in nominally zero Ca^2+^ (due to the presence of millimolar concentrations of Ca^2+^ chelators in the lysis buffers) [52,73,75,76]. Moreover, TIRFM experiments demonstrated that after ER Ca^2+^ depletion, SARAF co-localizes with STIM1 at ER-PM junctions in cells pre-loaded with BAPTA and that the SARAF E148A mutant, which does not induce SOCE inhibition, maintains interaction with STIM1 [52]. Therefore, the interaction of SARAF with either the resting or activated forms of STIM1 does not require high levels of Ca^2+^ and is not strictly correlated with channel inhibition.

**Figure 2:**
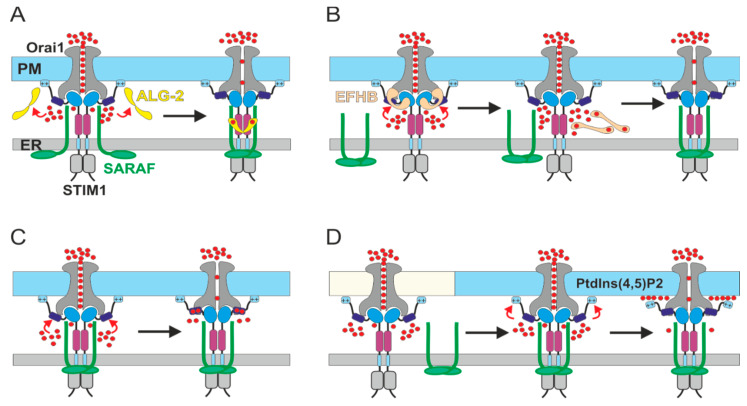
Models for SCDI by SARAF. Rectangle and oval symbols indicate the different domains of STIM1 as in Figure 1. (**A**) Ca^2+^-dependent interaction between ALG-2 (yellow) and SARAF (green) promotes channel inactivation via stabilization of SARAF dimers. (**B**) EFHB (brown) binds to the STIM1-Orai1 channel complex. Increase in Ca^2+^ causes disassociation of EFHB from STIM1. Subsequent binding of SARAF to STIM1 promotes channel inactivation. (**C**) Binding of Ca^2+^ to the STIM1 ID induces persistent channel inactivation when SARAF interacts with STIM1. (**D**) Binding of SARAF to STIM1 facilitates translocation of the channel complex from PM microdomains poor with PtdIns(4,5)P2 (off-white) to domains enriched with PtdIns(4,5)P2 (cyan). Local increase in Ca^2+^ promotes channel inactivation by remodeling the interaction of STIM1 with the PM.

What is then the mechanism through which SARAF promotes CRAC channel SCDI? As mentioned above, the answer is not known but there is evidence to support several alternatives (Figure 2).

One option is that SARAF cooperates with a Ca^2+^-binding protein to promote channel inhibition. There are several potential Ca^2+^ binding candidates. In the first scenario, interaction between SARAF and the penta-EF-hand Ca^2+^-binding protein ALG-2 may promote SCDI (Figure 2A). In support of such a scenario, Kimberlin et al. have shown that dimerization of SARAF could enhance SOCE inhibition [74] and Zhang et al. showed that ALG-2 physically interacts with the cytosolic-facing domain of SARAF and stabilizes dimerization of this region in a Ca^2+^-dependent manner [90]. While it is currently unknown if ALG-2 indeed modulates SOCE, a number of other Ca^2+^-binding proteins, including CRACR2A [91], calmodulin [56], and EFHB [75] have been shown to do so. Whether SARAF cooperates with either calmodulin or CRACR2A for inhibition of SOCE is not known. Conversely, a recent study showed that modulation of EFHB expression affects both SOCE and the interaction between STIM1 and SARAF [75]. The study demonstrated that ER Ca^2+^ depletion in HeLa cells elicits transient binding of EFHB to STIM1 in parallel with unbinding of SARAF from STIM1 and that preloading of cells with BAPTA stabilizes an interaction between EFHB and STIM1. Based on these findings, a model was presented in which EFHB contributes to SCDI by competing with SARAF over binding to STIM1 in a Ca^2+^-dependent manner (Figure 2B). The transient and reversible disassociation of SARAF and STIM1 after ER Ca^2+^ depletion is consistent with data from additional studies [37,76]. However, the idea that STIM1 and SARAF re-associate after ER Ca^2+^ depletion only when cytosolic Ca^2+^ rises is inconsistent with data from other studies which show that SARAF and STIM1 interact after store depletion and under low levels of free Ca^2+^ [37,52,63,73], that the interaction is also maintained in cells loaded with BAPTA and is unaffected by intracellular Ca^2+^ rise following induction of SOCE [52]. Further work that examines whether and how EFHB affects the CRAC current is required to shed light on this interesting possibility. A second option is that Ca^2+^ binding directly to SARAF or STIM1 operates to switch a non-inhibitory interaction between SARAF and STIM1 into an inhibitory one (Figure 2C). SARAF has no identifiable Ca^2+^ binding sites. On the other hand, the STIM1 ID region has been shown to bind to Ca^2+^ [92] and to affect CRAC channel CDI by SARAF, suggesting that this region in STIM1 could function as a Ca^2+^ sensor for SCDI [37]. A third alternative is that binding of SARAF to STIM1 is not inhibitory on its own but instead serves to direct the STIM1-Orai1 channel complex to specialized ER-PM contact sites where SCDI takes place (Figure 2D). Several studies have demonstrated a critical role for membrane lipids in the regulation of CRAC channel function [63,83,93,94,95,96]. An important report by Maléth et al. showed that upon ER Ca^2+^ depletion, STIM1 translocates initially to ER-PM sites poor with phosphatidylinositol 4,5-bisphosphate (PtdIns(4,5)P2) and that it later migrates to ones enriched with PtdIns(4,5)P2 [63]. The composition of PtdIns(4,5)P2-poor microdomains is currently unknown but likely includes PtdIns4P or PtdIn3P, which have been known to impact CRAC channel activation [83,97,98,99]. Notably, while depletion of PtdIns(4,5)P2 did not alter the degree of CRAC channel activation [63,83], it did reduce SCDI [63]. This finding suggests that SCDI occurs exclusively at ER-PM contact sites enriched with PtdIns(4,5)P2. To further test this idea, Maléth et al. replaced the C-terminal lipid-binding region of STIM1 with that of either Kras, that binds to PtdIns(4,5)P2, or that of Lyn or Hras that do not [100]. The different lipid-binding domains trapped STIM1 at ER-PM sites with different lipid compositions and enabled analyses of the effect of each lipid environment on SCDI and on interaction of STIM1 with SARAF. The results showed that when STIM1 was targeted to PtdIns(4,5)P2-rich domains, it interacted with SARAF and was sensitive to SCDI but not when it was targeted to domains poor with PtdIns(4,5)P2. Therefore, binding of SARAF to STIM1 at PtdIns(4,5)P2-poor microdomains may promote SCDI by facilitating the translocation of the STIM1-Orai1 channel complex to PtdIns(4,5)P2-rich microdomains. Interestingly, formation of Ca^2+^-phosphoinositide complexes alters the shape of phospholipid membranes and elevated levels of Ca^2+^ were found to interfere with lipid binding in a number of PtdIns(4,5)P2-binding proteins [101,102,103,104], including STIM2 [69]. Hence, an attractive feature of the phospholipid translocation model is that it also points towards a potential Ca^2+^-sensing mechanism for SCDI that involves Ca^2+^-dependent remodeling of the PtdIns(4,5)P2-rich microdomain. Whether binding of SARAF to STIM1 facilitates the translocation of the STIM1-Orai1 channel complex to ER-PM domains enriched with PtdIns(4,5)P2 and whether elevated Ca^2+^ modulates STIM1-phospholipid interaction are currently unknown and await future investigation.

## 7. The Regulatory Function of the ER Luminal-Facing Domain of SARAF

**Figure 3:**
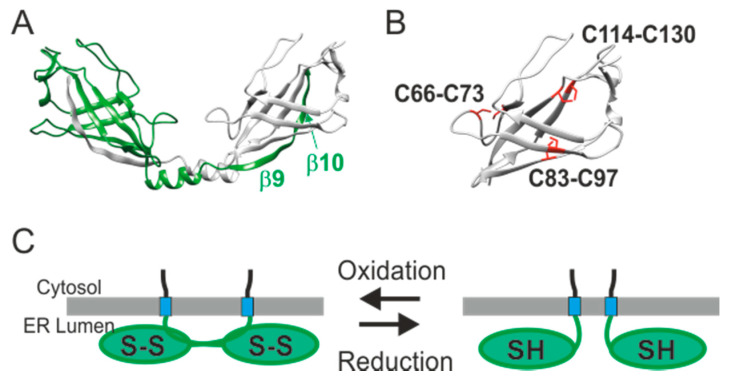
Structure and putative redox-dependent rearrangement of the SARAF luminal fold. (**A**) Structure of the SARAF domain-swapped dimer (PDB ID: 6O2U). Chains A and B are shown in green and grey, respectively, and the 9th and 10th β-strands from chain A are also labeled. (**B**) Magnification of the structure of b1 to b8 from chain A with cysteine residues forming disulfide bonds highlighted in red. (**C**) Cartoon representation of the SARAF luminal fold showing putative dimer to monomer reversible transitions mediated by oxidation-reduction of cysteine pairs indicated in (**B**). S–S denotes disulfide bonds.

While inhibition of CRAC channel by SARAF does not require the ER luminal-facing region of the protein, the degree of inhibition is modulated by this domain [52]. Recently, a study by the Reuveny and Minor groups reported the X-ray crystal structure of the SARAF luminal domain [74]. The structure shows that the SARAF luminal domain forms dimers (Figure 3A). Each monomer contains a 10-stranded β-sandwich fold constrained by a set of three disulfide bonds which establishes the “SARAF fold” (Figure 3A,B). The SARAF dimer is formed by the “SARAF luminal switch”—a domain swap arrangement in which the last two β-strands (β9 and β10) from each subunit are exchanged. Initial characterization of the effect of SARAF on CRAC channel deactivation suggested that SARAF somehow senses changes in luminal Ca^2+^ levels [52]. However, although the SARAF luminal domain was crystallized in 1 mM Ca^2+^, no Ca^2+^ binding sites were resolved therein [74]. The finding that the SARAF fold is stabilized by three conserved sets of disulfide bonds is highly interesting and indicates that one or more of the three cysteine pairs could operate as a redox sensor. Given that redox and Ca^2+^ are tightly coupled in the ER lumen [105,106,107], changes in ER Ca^2+^ that induce changes in the ER redox state could affect the oligomeric state of SARAF through reversible disulfide bond formation (Figure 3C). An indication that the oligomeric state of SARAF is indeed dynamic was shown by FRET experiments in live cells [74]. Therefore, an intriguing possibility is that dynamic changes in ER redox modulate the oligomeric state of the SARAF luminal fold and thus affect the action of the SOAR binding C-terminal region. Consistent with this idea, following transient ER Ca^2+^ depletion and under conditions that allow for ER Ca^2+^ refilling in cells, currents recorded in cells expressing a monomeric SARAF mutant lacking the SARAF luminal switch (SARAF Δ150–165) were transiently larger than those in cells expressing the full length protein [74]. The underlying mechanism behind the effect, however, is presently unclear and alternative scenarios must also be considered. The results could suggest that the dimeric state of SARAF accelerates CRAC channel deactivation; however, considering the effect of SARAF on channel activation [76,77], the results could alternatively imply that monomeric SARAF is more efficient in promoting CRAC channel activation than the SARAF dimer. Overall, while the luminal-facing domain of SARAF clearly affects CRAC channel function, remaining important questions are whether and how redox changes in the ER lumen induce oligomeric changes in SARAF and how such changes affect CRAC channel function.

## 8. The Physiological Role of SARAF

Consistent with its role in the regulation of SOCE, the expression of SARAF appears in a wide range of cell and tissue types similar to those of STIM1 or Orai1 [108,109,110]. Moreover, similar to STIM1 and Orai1, the expression of SARAF has also been shown to be regulated by androgen receptor stimulation [108,111,112]. Although changes in SARAF transcripts or protein levels have been reported in a number of pathologies, including pancreatitis [113], cancer [111,114], and neurodegenerative [114,115,116] or cardiovascular [117,118,119,120,121] diseases, there is no direct indication that loss- or gain-of-function mutations in SARAF lead to any specific pathophysiology at this time.

Several activating mutations in STIM1 or Orai1 are known to cause the rare Stormorken syndrome, whose features include congenital miosis and tubular aggregate myopathy. Interestingly, many of the disease-causing mutations are gain-of-function mutations that induce spontaneous channel activation. Functional analyses of these mutations showed that SCDI is perturbed in some cases [57], although not in all [122], suggesting that deficiency in SCDI may contribute to the disease state. As mentioned above, whether changes in SARAF expression or loss-of-function mutations in SARAF are associated with Stormorken syndrome is currently unknown but presents an interesting avenue to explore.

Studies in mice lacking SARAF expression provided new insights regarding the physiological roles of SARAF [113,120]. The reports have shown that mice with whole body knockout of SARAF are viable and appear to develop normally, indicating that loss of SARAF is overall well tolerated. It is worth mentioning that this finding is not very surprising given that the contribution of SARAF to regulation of Ca^2+^ entry in cells is likely to be compensated for by a number of alternative mechanisms. Nevertheless, the loss of SARAF expression is not without effect and at the cellular level leads to perturbed Ca^2+^ homeostasis in pancreatic acini cells [113], though not in cardiomyocytes [120], while at the tissue level in either heart or pancreas, the effect of SARAF deletion on function appears predominantly under pathological conditions [113,120]. In the following sections we discuss the role of SARAF under pathological states of cardiovascular, pancreatic and smooth muscle tissues.

## 9. The Role of SARAF in Pathologies of Cardiovascular Tissues

Elevated expression levels of SARAF were reported in patients with dilated cardiomyopathy (DCM), suggesting a possible role for SARAF in cardiac pathology associated with sustained hypertrophy [119]. The role of SARAF in cardiac hypertrophy, however, yielded apparently conflicting findings. Suggesting that increased expression of SARAF in the heart is not pathological and could play a protective role, Dai et al. showed that mice treated with SARAF lentiviruses exhibit normal cardiac size and function despite a significant increase in the expression of SARAF [118]. Notably, overexpression of SARAF in the heart blunted the increase in ventricle wall size after abdominal aortic constriction [118]. In contrast, using a different experimental model for pressure overload, Sanlialp et al. reported that full-body knockout of SARAF expression diminished cardiac hypertrophy following transverse aortic constriction (TAC) and that adeno-associated viruses induced overexpression of SARAF in the heart that contributed to pathological cell growth following treatment with Angiotensin II (Ang II) [120]. It is possible that the differences in the experimental models employed in the above reports contributed to the different effects of SARAF on hypertrophy responses. Moreover, considering the tight relationship between angiogenesis and pathological cardiac hypertrophy, it is worth mentioning that SARAF was shown to contribute to different steps in the angiogenesis process [77]. Hence, since overexpression of SARAF was driven by cardiomyocyte-specific myosin light chain ventricular isoform (MLC-2v) promoter only in one of the two studies [120], cell-type specific functions of SARAF may have also contributed to the observed differences. At the cellular level, the effects of SARAF on cardiomyocyte cell growth were associated with changes in calcium and mTORC1 signaling and were partially dependent on STIM1 expression, revealing a novel role for SARAF in regulating the crosstalk between SR calcium homeostasis and mTORC1 signaling [120]. Interestingly, several of the phenotypes of mice with SARAF deletion or overexpression are similar to those observed in animal models with altered expression of STIM1. While deletion of either STIM1 or SARAF in the heart protects against pressure-induced hypertrophy [120,123], overexpression of either protein leads to an increase in the magnitude and frequency of Ca^2+^ transient currents and also to similar hypertrophic responses following TAC, including increased heart weight and worsened remodeling of cardiac function [120,124]. These findings therefore suggest that in the heart, STIM1 and SARAF undergo a cooperative but not antagonizing interaction. The positive contribution of SARAF, STIM1, and Orai1 to both store-operated Ca^2+^ entry in endothelial cells and to angiogenesis [77,88,89,125] suggests that such a cooperative relationship between SARAF and CRAC channels may also occur in additional cell types and tissues.

## 10. The Role of SARAF in Acute Pancreatitis

Aberrant calcium signaling is a hallmark of pancreatic inflammation [126,127] and SOCE mediated by either Orai1 [128,129,130] or TRPC1 and TRPC3 [131,132,133,134,135] channels is the main Ca^2+^ entry pathway in pancreatic duct and acinar cells. Notably, SARAF inhibits the activity of either Orai1 [37,52,63,73,76] or TRPC1 [72] channels, suggesting it may play a key role in Ca^2+^ signaling in the pancreas. The potential therapeutic benefit of SOCE inhibition by SARAF, together with the finding that SARAF expression in the pancreas is reduced in patients with pancreatitis, prompted Son et al. to investigate a possible role of SARAF during acute pancreatitis [113]. In vitro studies utilizing cells with deleted or elevated expression of SARAF revealed a critical role for SARAF in SOCE inhibition in pancreatic acinar cells. Curiously, however, interaction between SARAF and STIM1 in either HEK293 or mouse pancreatic acini cells increased during the first couple of minutes following stimulation with carbachol and remained stable when cells were stimulated with low levels of the agonist but gradually diminished at high levels thereof. The latter decrease in SARAF-STIM1 interaction is somewhat surprising considering that it is not observed after ER Ca^2+^ depletion with SERCA inhibitors [52,63] or after induction of SOCE [52] and since stimulation at high agonist levels is likely to produce a stronger intracellular Ca^2+^ response followed by a more robust SCDI than those with low levels of the agonist. Although the cause for this decrease was not elucidated, a possible explanation is that it results from strong phospholipase C (PLC)-induced depletion of PtdIns(4,5)P2 in the cell membrane, which in turn diminishes SCDI by SARAF [63]. Remarkably, in vivo studies showed that the deletion of SARAF expression led to high basal pancreatic trypsin activity and to elevated secretion of amylase and saliva upon muscarinic receptor stimulation. Importantly, induction of acute pancreatitis by low doses of caerulein led to a more aggravated response in SARAF KO mice compared to WT mice whereas SARAF over-expressing mice exhibited a more protective effect, suggesting that SOCE inhibition by SARAF plays a critical protective role during acute pancreatitis.

## 11. The Role of SARAF in Smooth Muscle Remodeling

In both vascular and airway tissues, the remodeling of smooth muscle cells (SMC) is considered to be an important mechanism that underlies different disease states, including restenosis, atherosclerosis, hypertension, and atopic asthma [136]. SMC remodeling is characterized by a phenotypic change in which cells transition from a normal contractile phenotype to a more migratory-proliferating one and the process is associated with upregulation of Ca^2+^ entry via STIM and Orai. Interestingly, in both airway and vascular SMCs, STIM and Orai establish two distinct types of channels, store-operated CRAC channels and store-independent arachidonate-regulated Ca^2+^ (ARC) channels. Both CRAC and ARC channels have been shown to be regulated by SARAF in other cell types [52,71]; however, whether SARAF exerts a similar effect on both types of channels in SMCs is not firmly established yet. Nonetheless, accumulating evidences suggest that SARAF plays an important role during pathological remodeling of both types of smooth muscle tissues [108,117,137]. Using a balloon-induced carotid artery injury model in rats, Yang et al. showed that while in healthy arteries the expression of SARAF was significant in the media and intima layers, it was very low in the newly formed neointima layer in injured arteries. Importantly, the study showed that increased levels of SARAF may endow protection against neointimal hyperplasia. Overexpression of SARAF in cultured vascular SMC-attenuated PDGF evoked intracellular Ca^2+^ responses and suppressed cellular proliferation and migration. Likewise, overexpression of SARAF in vivo partially suppressed neointima formation after balloon injury. Studies in airway SMC imply a similar protective role for SARAF against inflammation-induced SMC remodeling. In human airway SMCs, exposure to the pro-inflammatory cytokine TNF or treatment with SARAF siRNAs caused reduction in expression of SARAF and elevation in SOCE [108,137]. Likewise, the SARAF-deficient cells exhibited increased migration and proliferation [137]. Using a model for asthma in mice, Wang et al. showed that remodeling of SMCs after either acute or chronic inflammation in the lungs was correlated with reduction in expression of SARAF and that both severity of inflammation as well as SMC remodeling was significantly reduced in mice over-expressing SARAF. Therefore, through its suppressive effects on Ca^2+^ entry in SMC, SARAF appears to play an important protective role during pathological remodeling of smooth muscle tissue.

## 12. Concluding Remarks and Future Directions

By shaping cellular Ca^2+^ entry in cells, slow CDI of CRAC channels plays key roles in regulation of gene expression, secretion, and cellular toxicity [113,138]. As the inactivation process involves different mechanisms in different types of cells, understanding the molecular basis for this functional heterogeneity represents a significant challenge for future work. The discovery of SARAF and its role in regulation of CRAC channel function constitutes a step forward in this direction. It enabled a first examination of the role of SCDI in vivo and confirmed that SCDI indeed plays a protective role against Ca^2+^-induced cellular toxicity [76] or pathological tissue remodeling [117,137]. However, in some tissues, the role of SARAF appears to extend beyond its contribution to SCDI [77,120], thus calling for further examination of the function of SARAF in additional tissues and under different physiological and pathophysiological states. There are also a number of key issues that require elucidation from a mechanistic point of view. The regulatory effect of SARAF on CRAC channel function depends on multiple factors, including the levels of free Ca^2+^ in the cytosol, the oligomeric state of the SARAF luminal fold, and the localization of the channel at PtdIns(4,5)P2-rich PM microdomains. The mechanism by which changes in these factors are communicated via SARAF to the STIM1-Orai1 channel complex remains elusive. This issue segues with the final point as there exists strong evidence that the increase in interaction between SARAF and STIM1 correlates with CRAC channel activation but not SCDI and that under some circumstances SARAF promotes rather than inhibits Ca^2+^ entry in cells [76,77]. Therefore, exactly how interaction between SARAF and the STIM1-Orai1 channel complex affects either the process of activation or inactivation is another fundamental issue that awaits elucidation.

## Figures and Tables

**Table 1:** Summary of differences in properties of SCDI in Jurkat, RBL, and HEK293 cells.

SCDI Properties	Jurkat T-lymphocytes	Rat Basophilic Leukemia Cells	Human Embryonic Kidney Cells
Distance from CRAC channel	~10–50 nm [59] or >100 nm [53]	>100 nm [54,60]	~10–50 nm [57,61]
Ca^2+^ IC50	?	~0.5 µM [62]	~0.2 µM [61]
Recovery from inactivation	Full current recovery [53]	No current recovery [54]	Intermediate current recovery [52,61]
Role of regulators	Promoted by SARAF [52] and ORMDL3 [55]	Insensitive to CaM [54]	Promoted by SARAF [37,52,63] and CaM [56]

## References

[1] Putney J.W. (1986). A Model for Receptor-Regulated Calcium Entry. Cell Calcium.

[2] Lacruz R.S., Feske S. (2015). Diseases Caused by Mutations in ORAI1 and STIM1. Ann. N. Y. Acad. Sci..

[3] Prakriya M., Lewis R.S. (2015). Store-Operated Calcium Channels. Physiol. Rev..

[4] Ong H.L., de Souza L.B., Ambudkar I.S. (2016). Role of TRPC Channels in Store-Operated Calcium Entry. Adv. Exp. Med. Biol..

[5] Zhang S.L., Yeromin A.V., Zhang X.H.-F., Yu Y., Safrina O., Penna A., Roos J., Stauderman K.A., Cahalan M.D. (2006). Genome-Wide RNAi Screen of Ca^2+^ Influx Identifies Genes That Regulate Ca^2+^ Release-Activated Ca^2+^ Channel Activity. Proc. Natl. Acad. Sci. USA.

[6] Zhang S.L., Yu Y., Roos J., Kozak J.A., Deerinck T.J., Ellisman M.H., Stauderman K.A., Cahalan M.D. (2005). STIM1 Is a Ca^2+^ Sensor That Activates CRAC Channels and Migrates from the Ca^2+^ Store to the Plasma Membrane. Nature.

[7] Roos J., DiGregorio P.J., Yeromin A.V., Ohlsen K., Lioudyno M., Zhang S., Safrina O., Kozak J.A., Wagner S.L., Cahalan M.D. (2005). STIM1, an Essential and Conserved Component of Store-Operated Ca^2+^ Channel Function. J. Cell Biol..

[8] Yeromin A.V., Zhang S.L., Jiang W., Yu Y., Safrina O., Cahalan M.D. (2006). Molecular Identification of the CRAC Channel by Altered Ion Selectivity in a Mutant of Orai. Nature.

[9] Feske S., Gwack Y., Prakriya M., Srikanth S., Puppel S.-H., Tanasa B., Hogan P.G., Lewis R.S., Daly M., Rao A. (2006). A Mutation in Orai1 Causes Immune Deficiency by Abrogating CRAC Channel Function. Nature.

[10] Prakriya M., Feske S., Gwack Y., Srikanth S., Rao A., Hogan P.G. (2006). Orai1 Is an Essential Pore Subunit of the CRAC Channel. Nature.

[11] Vig M., Peinelt C., Beck A., Koomoa D.L., Rabah D., Koblan-Huberson M., Kraft S., Turner H., Fleig A., Penner R. (2006). CRACM1 Is a Plasma Membrane Protein Essential for Store-Operated Ca^2+^ Entry. Science.

[12] Hoth M., Niemeyer B.A. (2013). The Neglected CRAC Proteins: Orai2, Orai3, and STIM2. Curr. Top. Membr..

[13] Hou X., Pedi L., Diver M.M., Long S.B. (2012). Crystal Structure of the Calcium Release-Activated Calcium Channel Orai. Science.

[14] Liu X., Wu G., Yu Y., Chen X., Ji R., Lu J., Li X., Zhang X., Yang X., Shen Y. (2019). Molecular Understanding of Calcium Permeation through the Open Orai Channel. PLoS Biol..

[15] Palty R., Stanley C., Isacoff E.Y. (2015). Critical Role for Orai1 C-Terminal Domain and TM4 in CRAC Channel Gating. Cell Res..

[16] Li Z., Lu J., Xu P., Xie X., Chen L., Xu T. (2007). Mapping the Interacting Domains of STIM1 and Orai1 in Ca^2+^ Release-Activated Ca^2+^ Channel Activation. J. Biol. Chem..

[17] Palty R., Isacoff E.Y. (2016). Cooperative Binding of Stromal Interaction Molecule 1 (STIM1) to the N and C Termini of Calcium Release-Activated Calcium Modulator 1 (Orai1). J. Biol. Chem..

[18] McNally B.A., Somasundaram A., Jairaman A., Yamashita M., Prakriya M. (2013). The C- and N-Terminal STIM1 Binding Sites on Orai1 Are Required for Both Trapping and Gating CRAC Channels. J. Physiol..

[19] Gudlur A., Quintana A., Zhou Y., Hirve N., Mahapatra S., Hogan P.G. (2014). STIM1 Triggers a Gating Rearrangement at the Extracellular Mouth of the ORAI1 Channel. Nat. Commun..

[20] Derler I., Plenk P., Fahrner M., Muik M., Jardin I., Schindl R., Gruber H.J., Groschner K., Romanin C. (2013). The Extended Transmembrane Orai1 N-Terminal (ETON) Region Combines Binding Interface and Gate for Orai1 Activation by STIM1*♦. J. Biol. Chem..

[21] Lis A., Zierler S., Peinelt C., Fleig A., Penner R. (2010). A Single Lysine in the N-Terminal Region of Store-Operated Channels Is Critical for STIM1-Mediated Gating. J. Gen. Physiol..

[22] Zheng H., Zhou M.-H., Hu C., Kuo E., Peng X., Hu J., Kuo L., Zhang S.L. (2013). Differential Roles of the C and N Termini of Orai1 Protein in Interacting with Stromal Interaction Molecule 1 (STIM1) for Ca^2+^ Release-Activated Ca^2+^ (CRAC) Channel Activation. J. Biol. Chem..

[23] Liou J., Kim M.L., Heo W.D., Jones J.T., Myers J.W., Ferrell J.E., Meyer T. (2005). STIM Is a Ca^2+^ Sensor Essential for Ca^2+^-Store-Depletion-Triggered Ca^2+^ Influx. Curr. Biol..

[24] Gudlur A., Zeraik A.E., Hirve N., Rajanikanth V., Bobkov A.A., Ma G., Zheng S., Wang Y., Zhou Y., Komives E.A. (2018). Calcium Sensing by the STIM1 ER-Luminal Domain. Nat. Commun..

[25] Luik R.M., Wang B., Prakriya M., Wu M.M., Lewis R.S. (2008). Oligomerization of STIM1 Couples ER Calcium Depletion to CRAC Channel Activation. Nature.

[26] Ma G., Wei M., He L., Liu C., Wu B., Zhang S.L., Jing J., Liang X., Senes A., Tan P. (2015). Inside-out Ca^2+^ Signalling Prompted by STIM1 Conformational Switch. Nat. Commun..

[27] Zhou Y., Srinivasan P., Razavi S., Seymour S., Meraner P., Gudlur A., Stathopulos P.B., Ikura M., Rao A., Hogan P.G. (2013). Initial Activation of STIM1, the Regulator of Store-Operated Calcium Entry. Nat. Struct. Mol. Biol..

[28] Hirve N., Rajanikanth V., Hogan P.G., Gudlur A. (2018). Coiled-Coil Formation Conveys a STIM1 Signal from ER Lumen to Cytoplasm. Cell Rep..

[29] Fahrner M., Muik M., Schindl R., Butorac C., Stathopulos P., Zheng L., Jardin I., Ikura M., Romanin C. (2014). A Coiled-Coil Clamp Controls Both Conformation and Clustering of Stromal Interaction Molecule 1 (STIM1). J. Biol. Chem..

[30] Fahrner M., Stadlbauer M., Muik M., Rathner P., Stathopulos P., Ikura M., Müller N., Romanin C. (2018). A Dual Mechanism Promotes Switching of the Stormorken STIM1 R304W Mutant into the Activated State. Nat. Commun..

[31] Muik M., Fahrner M., Derler I., Schindl R., Bergsmann J., Frischauf I., Groschner K., Romanin C. (2009). A Cytosolic Homomerization and a Modulatory Domain within STIM1 C Terminus Determine Coupling to ORAI1 Channels. J. Biol. Chem..

[32] Muik M., Fahrner M., Schindl R., Stathopulos P., Frischauf I., Derler I., Plenk P., Lackner B., Groschner K., Ikura M. (2011). STIM1 Couples to ORAI1 via an Intramolecular Transition into an Extended Conformation. EMBO J..

[33] Muik M., Frischauf I., Derler I., Fahrner M., Bergsmann J., Eder P., Schindl R., Hesch C., Polzinger B., Fritsch R. (2008). Dynamic Coupling of the Putative Coiled-Coil Domain of ORAI1 with STIM1 Mediates ORAI1 Channel Activation. J. Biol. Chem..

[34] Korzeniowski M.K., Manjarrés I.M., Varnai P., Balla T. (2010). Activation of STIM1-Orai1 Involves an Intramolecular Switching Mechanism. Sci. Signal..

[35] Yuan J.P., Zeng W., Dorwart M.R., Choi Y.-J., Worley P.F., Muallem S. (2009). SOAR and the Polybasic STIM1 Domains Gate and Regulate Orai Channels. Nat. Cell Biol..

[36] Park C.Y., Hoover P.J., Mullins F.M., Bachhawat P., Covington E.D., Raunser S., Walz T., Garcia K.C., Dolmetsch R.E., Lewis R.S. (2009). STIM1 Clusters and Activates CRAC Channels via Direct Binding of a Cytosolic Domain to Orai1. Cell.

[37] Jha A., Ahuja M., Maléth J., Moreno C.M., Yuan J.P., Kim M.S., Muallem S. (2013). The STIM1 CTID Domain Determines Access of SARAF to SOAR to Regulate Orai1 Channel Function. J. Cell Biol..

[38] Lee S.K., Lee M., Jeong S.J., Qin X., Lee A.R., Park H., Park C.Y. (2019). IDstim Helps STIM1 Keep Inactive via Intramolecular Binding to the Coiled-Coil Domain in a Resting State. J. Cell Sci..

[39] Luik R.M., Wu M.M., Buchanan J., Lewis R.S. (2006). The Elementary Unit of Store-Operated Ca^2+^ Entry: Local Activation of CRAC Channels by STIM1 at ER-Plasma Membrane Junctions. J. Cell Biol..

[40] Wu M.M., Buchanan J., Luik R.M., Lewis R.S. (2006). Ca^2+^ Store Depletion Causes STIM1 to Accumulate in ER Regions Closely Associated with the Plasma Membrane. J. Cell Biol..

[41] Xu P., Lu J., Li Z., Yu X., Chen L., Xu T. (2006). Aggregation of STIM1 underneath the Plasma Membrane Induces Clustering of Orai1. Biochem. Biophys. Res. Commun..

[42] Berridge M.J., Bootman M.D., Lipp P. (1998). Calcium—A Life and Death Signal. Nature.

[43] Jousset H., Frieden M., Demaurex N. (2007). STIM1 Knockdown Reveals That Store-Operated Ca^2+^ Channels Located Close to Sarco/Endoplasmic Ca^2+^ ATPases (SERCA) Pumps Silently Refill the Endoplasmic Reticulum. J. Biol. Chem..

[44] Malli R., Naghdi S., Romanin C., Graier W.F. (2008). Cytosolic Ca^2+^ Prevents the Subplasmalemmal Clustering of STIM1: An Intrinsic Mechanism to Avoid Ca^2+^ Overload. J. Cell Sci..

[45] Smyth J.T., Dehaven W.I., Bird G.S., Putney J.W. (2008). Ca^2+^-Store-Dependent and -Independent Reversal of Stim1 Localization and Function. J. Cell Sci..

[46] Bird G.S., Hwang S.-Y., Smyth J.T., Fukushima M., Boyles R.R., Putney J.W. (2009). STIM1 Is a Calcium Sensor Specialized for Digital Signaling. Curr. Biol..

[47] Derler I., Fahrner M., Muik M., Lackner B., Schindl R., Groschner K., Romanin C. (2009). A Ca^2+^ Release-Activated Ca^2+^ (CRAC) Modulatory Domain (CMD) within STIM1 Mediates Fast Ca^2+^-Dependent Inactivation of ORAI1 Channels. J. Biol. Chem..

[48] Mullins F.M., Yen M., Lewis R.S. (2016). Orai1 Pore Residues Control CRAC Channel Inactivation Independently of Calmodulin. J. Gen. Physiol..

[49] Zweifach A., Lewis R.S. (1995). Rapid Inactivation of Depletion-Activated Calcium Current (ICRAC) Due to Local Calcium Feedback. J. Gen. Physiol..

[50] Mullins F.M., Lewis R.S. (2016). The Inactivation Domain of STIM1 Is Functionally Coupled with the Orai1 Pore to Enable Ca^2+^-Dependent Inactivation. J. Gen. Physiol..

[51] Lee K.P., Yuan J.P., Zeng W., So I., Worley P.F., Muallem S. (2009). Molecular Determinants of Fast Ca^2+^-Dependent Inactivation and Gating of the Orai Channels. Proc. Natl. Acad. Sci. USA.

[52] Palty R., Raveh A., Kaminsky I., Meller R., Reuveny E. (2012). SARAF Inactivates the Store Operated Calcium Entry Machinery to Prevent Excess Calcium Refilling. Cell.

[53] Zweifach A., Lewis R.S. (1995). Slow Calcium-Dependent Inactivation of Depletion-Activated Calcium Current. Store-Dependent and -Independent Mechanisms. J. Biol. Chem..

[54] Parekh A.B. (1998). Slow Feedback Inhibition of Calcium Release-Activated Calcium Current by Calcium Entry. J. Biol. Chem..

[55] Carreras-Sureda A., Cantero-Recasens G., Rubio-Moscardo F., Kiefer K., Peinelt C., Niemeyer B.A., Valverde M.A., Vicente R. (2013). ORMDL3 Modulates Store-Operated Calcium Entry and Lymphocyte Activation. Hum. Mol. Genet..

[56] Li X., Wu G., Yang Y., Fu S., Liu X., Kang H., Yang X., Su X.-C., Shen Y. (2017). Calmodulin Dissociates the STIM1-Orai1 Complex and STIM1 Oligomers. Nat. Commun..

[57] Nesin V., Wiley G., Kousi M., Ong E.-C., Lehmann T., Nicholl D.J., Suri M., Shahrizaila N., Katsanis N., Gaffney P.M. (2014). Activating Mutations in STIM1 and ORAI1 Cause Overlapping Syndromes of Tubular Myopathy and Congenital Miosis. Proc. Natl. Acad. Sci. USA.

[58] Louzao M.C., Ribeiro C.M., Bird G.S., Putney J.W. (1996). Cell Type-Specific Modes of Feedback Regulation of Capacitative Calcium Entry. J. Biol. Chem..

[59] Montalvo G.B., Artalejo A.R., Gilabert J.A. (2006). ATP from Subplasmalemmal Mitochondria Controls Ca^2+^-Dependent Inactivation of CRAC Channels. J. Biol. Chem..

[60] Gilabert J.A., Parekh A.B. (2000). Respiring Mitochondria Determine the Pattern of Activation and Inactivation of the Store-Operated Ca^2+^ Current I(CRAC). EMBO J..

[61] Lis A., Peinelt C., Beck A., Parvez S., Monteilh-Zoller M., Fleig A., Penner R. (2007). CRACM1, CRACM2, and CRACM3 Are Store-Operated Ca^2+^ Channels with Distinct Functional Properties. Curr. Biol..

[62] Ng S.-W., Bakowski D., Nelson C., Mehta R., Almeyda R., Bates G., Parekh A.B. (2012). Cysteinyl Leukotriene Type I Receptor Desensitization Sustains Ca^2+^-Dependent Gene Expression. Nature.

[63] Maléth J., Choi S., Muallem S., Ahuja M. (2014). Translocation between PI(4,5)P2-Poor and PI(4,5)P2-Rich Microdomains during Store Depletion Determines STIM1 Conformation and Orai1 Gating. Nat. Commun..

[64] Naraghi M., Neher E. (1997). Linearized Buffered Ca^2+^ Diffusion in Microdomains and Its Implications for Calculation of [Ca^2+^] at the Mouth of a Calcium Channel. J. Neurosci..

[65] Bastián-Eugenio C.E., Bohórquez-Hernández A., Pacheco J., Sampieri A., Asanov A., Ocelotl-Oviedo J.P., Guerrero A., Darszon A., Vaca L. (2019). Heterologous Calcium-Dependent Inactivation of Orai1 by Neighboring TRPV1 Channels Modulates Cell Migration and Wound Healing. Commun. Biol..

[66] Liu Y., Zheng X., Mueller G.A., Sobhany M., DeRose E.F., Zhang Y., London R.E., Birnbaumer L. (2012). Crystal Structure of Calmodulin Binding Domain of Orai1 in Complex with Ca^2+^ Calmodulin Displays a Unique Binding Mode. J. Biol. Chem..

[67] Traxler L., Rathner P., Fahrner M., Stadlbauer M., Faschinger F., Charnavets T., Müller N., Romanin C., Hinterdorfer P., Gruber H.J. (2017). Detailed Evidence for an Unparalleled Interaction Mode between Calmodulin and Orai Proteins. Angew. Chem. Int. Ed. Engl..

[68] Bauer M.C., O’Connell D., Cahill D.J., Linse S. (2008). Calmodulin Binding to the Polybasic C-Termini of STIM Proteins Involved in Store-Operated Calcium Entry. Biochemistry.

[69] Bhardwaj R., Müller H.-M., Nickel W., Seedorf M. (2013). Oligomerization and Ca^2+^/Calmodulin Control Binding of the ER Ca^2+^-Sensors STIM1 and STIM2 to Plasma Membrane Lipids. Biosci. Rep..

[70] Albarran L., Regodón S., Salido G.M., Lopez J.J., Rosado J.A. (2017). Role of STIM1 in the Surface Expression of SARAF. Channels.

[71] Albarran L., Lopez J.J., Woodard G.E., Salido G.M., Rosado J.A. (2016). Store-Operated Ca^2+^ Entry-Associated Regulatory Factor (SARAF) Plays an Important Role in the Regulation of Arachidonate-Regulated Ca^2+^ (ARC) Channels. J. Biol. Chem..

[72] Albarran L., Lopez J.J., Gomez L.J., Salido G.M., Rosado J.A. (2016). SARAF Modulates TRPC1, but Not TRPC6, Channel Function in a STIM1-Independent Manner. Biochem. J..

[73] Lopez E., Frischauf I., Jardin I., Derler I., Muik M., Cantonero C., Salido G.M., Smani T., Rosado J.A., Redondo P.C. (2019). STIM1 Phosphorylation at Y316 Modulates Its Interaction with SARAF and the Activation of SOCE and ICRAC. J. Cell. Sci..

[74] Kimberlin C.R., Meshcheriakova A., Palty R., Raveh A., Karbat I., Reuveny E., Minor D.L. (2019). SARAF Luminal Domain Structure Reveals a Novel Domain-Swapped β-Sandwich Fold Important for SOCE Modulation. J. Mol. Biol..

[75] Albarran L., Lopez J.J., Jardin I., Sanchez-Collado J., Berna-Erro A., Smani T., Camello P.J., Salido G.M., Rosado J.A. (2018). EFHB Is a Novel Cytosolic Ca^2+^ Sensor That Modulates STIM1-SARAF Interaction. Cell. Physiol. Biochem..

[76] Albarran L., Lopez J.J., Amor N.B., Martin-Cano F.E., Berna-Erro A., Smani T., Salido G.M., Rosado J.A. (2016). Dynamic Interaction of SARAF with STIM1 and Orai1 to Modulate Store-Operated Calcium Entry. Sci. Rep..

[77] Galeano-Otero I., Del Toro R., Khatib A.-M., Rosado J.A., Ordóñez-Fernández A., Smani T. (2021). SARAF and Orai1 Contribute to Endothelial Cell Activation and Angiogenesis. Front. Cell Dev. Biol..

[78] Ma G., He L., Liu S., Xie J., Huang Z., Jing J., Lee Y.-T., Wang R., Luo H., Han W. (2020). Optogenetic Engineering to Probe the Molecular Choreography of STIM1-Mediated Cell Signaling. Nat. Commun..

[79] Grigoriev I., Gouveia S.M., van der Vaart B., Demmers J., Smyth J.T., Honnappa S., Splinter D., Steinmetz M.O., Putney J.W., Hoogenraad C.C. (2008). STIM1 Is a MT-plus-End-Tracking Protein Involved in Remodeling of the ER. Curr. Biol..

[80] Honnappa S., Gouveia S.M., Weisbrich A., Damberger F.F., Bhavesh N.S., Jawhari H., Grigoriev I., van Rijssel F.J.A., Buey R.M., Lawera A. (2009). An EB1-Binding Motif Acts as a Microtubule Tip Localization Signal. Cell.

[81] Liou J., Fivaz M., Inoue T., Meyer T. (2007). Live-Cell Imaging Reveals Sequential Oligomerization and Local Plasma Membrane Targeting of Stromal Interaction Molecule 1 after Ca^2+^ Store Depletion. Proc. Natl. Acad. Sci. USA.

[82] Ercan E., Momburg F., Engel U., Temmerman K., Nickel W., Seedorf M. (2009). A Conserved, Lipid-Mediated Sorting Mechanism of Yeast Ist2 and Mammalian STIM Proteins to the Peripheral ER. Traffic.

[83] Korzeniowski M.K., Popovic M.A., Szentpetery Z., Varnai P., Stojilkovic S.S., Balla T. (2009). Dependence of STIM1/Orai1-Mediated Calcium Entry on Plasma Membrane Phosphoinositides. J. Biol. Chem..

[84] Walsh C.M., Chvanov M., Haynes L.P., Petersen O.H., Tepikin A.V., Burgoyne R.D. (2009). Role of Phosphoinositides in STIM1 Dynamics and Store-Operated Calcium Entry. Biochem. J..

[85] Chen Y.-J., Chang C.-L., Lee W.-R., Liou J. (2017). RASSF4 Controls SOCE and ER-PM Junctions through Regulation of PI(4,5)P2. J. Cell Biol..

[86] Chang C.-L., Chen Y.-J., Quintanilla C.G., Hsieh T.-S., Liou J. (2018). EB1 Binding Restricts STIM1 Translocation to ER-PM Junctions and Regulates Store-Operated Ca^2+^ Entry. J. Cell Biol..

[87] Zheng S., Zhou L., Ma G., Zhang T., Liu J., Li J., Nguyen N.T., Zhang X., Li W., Nwokonko R. (2018). Calcium Store Refilling and STIM Activation in STIM- and Orai-Deficient Cell Lines. Pflug. Arch..

[88] Abdullaev I.F., Bisaillon J.M., Potier M., Gonzalez J.C., Motiani R.K., Trebak M. (2008). Stim1 and Orai1 Mediate CRAC Currents and Store-Operated Calcium Entry Important for Endothelial Cell Proliferation. Circ. Res..

[89] Qiu X., Dong K., Sun R. (2021). STIM1 Regulates Endothelial Calcium Overload and Cytokine Upregulation During Sepsis. J. Surg. Res..

[90] Zhang W., Muramatsu A., Matsuo R., Teranishi N., Kahara Y., Takahara T., Shibata H., Maki M. (2020). The Penta-EF-Hand ALG-2 Protein Interacts with the Cytosolic Domain of the SOCE Regulator SARAF and Interferes with Ubiquitination. Int. J. Mol. Sci..

[91] Srikanth S., Jung H.-J., Kim K.-D., Souda P., Whitelegge J., Gwack Y. (2010). A Novel EF-Hand Protein, CRACR2A, Is a Cytosolic Ca^2+^ Sensor That Stabilizes CRAC Channels in T Cells. Nat. Cell Biol..

[92] Mullins F.M., Park C.Y., Dolmetsch R.E., Lewis R.S. (2009). STIM1 and Calmodulin Interact with Orai1 to Induce Ca^2+^-Dependent Inactivation of CRAC Channels. Proc. Natl. Acad. Sci. USA.

[93] Derler I., Jardin I., Stathopulos P.B., Muik M., Fahrner M., Zayats V., Pandey S.K., Poteser M., Lackner B., Absolonova M. (2016). Cholesterol Modulates Orai1 Channel Function. Sci. Signal..

[94] Pacheco J., Dominguez L., Bohórquez-Hernández A., Asanov A., Vaca L. (2016). A Cholesterol-Binding Domain in STIM1 Modulates STIM1-Orai1 Physical and Functional Interactions. Sci. Rep..

[95] Bohórquez-Hernández A., Gratton E., Pacheco J., Asanov A., Vaca L. (2017). Cholesterol Modulates the Cellular Localization of Orai1 Channels and Its Disposition among Membrane Domains. Biochim. Biophys. Acta BBA Mol. Cell Biol. Lipids.

[96] Calloway N., Owens T., Corwith K., Rodgers W., Holowka D., Baird B. (2011). Stimulated Association of STIM1 and Orai1 Is Regulated by the Balance of PtdIns(4,5)P2 between Distinct Membrane Pools. J. Cell Sci..

[97] Rosado J.A., Sage S.O. (2000). Phosphoinositides Are Required for Store-Mediated Calcium Entry in Human Platelets. J. Biol. Chem..

[98] Watanabe H., Takahashi R., Zhang X.X., Kakizawa H., Hayashi H., Ohno R. (1996). Inhibition of Agonist-Induced Ca^2+^ Entry in Endothelial Cells by Myosin Light-Chain Kinase Inhibitor. Biochem. Biophys. Res. Commun..

[99] Broad L.M., Braun F.J., Lievremont J.P., Bird G.S., Kurosaki T., Putney J.W. (2001). Role of the Phospholipase C-Inositol 1,4,5-Trisphosphate Pathway in Calcium Release-Activated Calcium Current and Capacitative Calcium Entry. J. Biol. Chem..

[100] Heo W.D., Inoue T., Park W.S., Kim M.L., Park B.O., Wandless T.J., Meyer T. (2006). PI(3,4,5)P3 and PI(4,5)P2 Lipids Target Proteins with Polybasic Clusters to the Plasma Membrane. Science.

[101] Kang J.K., Kim O.-H., Hur J., Yu S.H., Lamichhane S., Lee J.W., Ojha U., Hong J.H., Lee C.S., Cha J.-Y. (2017). Increased Intracellular Ca^2+^ Concentrations Prevent Membrane Localization of PH Domains through the Formation of Ca^2+^-Phosphoinositides. Proc. Natl. Acad. Sci. USA.

[102] Bilkova E., Pleskot R., Rissanen S., Sun S., Czogalla A., Cwiklik L., Róg T., Vattulainen I., Cremer P.S., Jungwirth P. (2017). Calcium Directly Regulates Phosphatidylinositol 4,5-Bisphosphate Headgroup Conformation and Recognition. J. Am. Chem. Soc..

[103] Sarmento M.J., Coutinho A., Fedorov A., Prieto M., Fernandes F. (2014). Ca^2+^ Induces PI(4,5)P2 Clusters on Lipid Bilayers at Physiological PI(4,5)P2 and Ca^2+^ Concentrations. Biochim. Biophys. Acta.

[104] Graber Z.T., Shi Z., Baumgart T. (2017). Cations Induce Shape Remodeling of Negatively Charged Phospholipid Membranes. Phys. Chem. Chem. Phys..

[105] Avezov E., Cross B.C.S., Kaminski Schierle G.S., Winters M., Harding H.P., Melo E.P., Kaminski C.F., Ron D. (2013). Lifetime Imaging of a Fluorescent Protein Sensor Reveals Surprising Stability of ER Thiol Redox. J. Cell. Biol..

[106] Enyedi B., Várnai P., Geiszt M. (2010). Redox State of the Endoplasmic Reticulum Is Controlled by Ero1L-Alpha and Intraluminal Calcium. Antioxid. Redox Signal..

[107] Birk J., Meyer M., Aller I., Hansen H.G., Odermatt A., Dick T.P., Meyer A.J., Appenzeller-Herzog C. (2013). Endoplasmic Reticulum: Reduced and Oxidized Glutathione Revisited. J. Cell. Sci..

[108] Kalidhindi R.S.R., Katragadda R., Beauchamp K.L., Pabelick C.M., Prakash Y.S., Sathish V. (2019). Androgen Receptor-Mediated Regulation of Intracellular Calcium in Human Airway Smooth Muscle Cells. Cell Physiol. Biochem..

[109] Berry P.A., Birnie R., Droop A.P., Maitland N.J., Collins A.T. (2011). The Calcium Sensor STIM1 Is Regulated by Androgens in Prostate Stromal Cells. Prostate.

[110] Liu G., Honisch S., Liu G., Schmidt S., Alkahtani S., AlKahtane A.A., Stournaras C., Lang F. (2015). Up-Regulation of Orai1 Expression and Store Operated Ca^2+^ Entry Following Activation of Membrane Androgen Receptors in MCF-7 Breast Tumor Cells. BMC Cancer.

[111] Romanuik T.L., Ueda T., Le N., Haile S., Yong T.M.K., Thomson T., Vessella R.L., Sadar M.D. (2009). Novel Biomarkers for Prostate Cancer Including Noncoding Transcripts. Am. J. Pathol..

[112] Romanuik T.L., Wang G., Holt R.A., Jones S.J.M., Marra M.A., Sadar M.D. (2009). Identification of Novel Androgen-Responsive Genes by Sequencing of LongSAGE Libraries. BMC Genom..

[113] Son A., Ahuja M., Schwartz D.M., Varga A., Swaim W., Kang N., Maleth J., Shin D.M., Muallem S. (2019). Ca^2+^ Influx Channel Inhibitor SARAF Protects Mice From Acute Pancreatitis. Gastroenterology.

[114] Iżykowska K., Przybylski G.K., Gand C., Braun F.C., Grabarczyk P., Kuss A.W., Olek-Hrab K., Bastidas Torres A.N., Vermeer M.H., Zoutman W.H. (2017). Genetic Rearrangements Result in Altered Gene Expression and Novel Fusion Transcripts in Sézary Syndrome. Oncotarget.

[115] Taha S., Aljishi M., Alsharoqi I., Bakhiet M. (2015). Differential Upregulation of the Hypothetical Transmembrane Protein 66 (TMEM66) in Multiple Sclerosis Patients with Potential Inflammatory Response. Biomed. Rep..

[116] Twine N.A., Janitz K., Wilkins M.R., Janitz M. (2011). Whole Transcriptome Sequencing Reveals Gene Expression and Splicing Differences in Brain Regions Affected by Alzheimer’s Disease. PLoS ONE.

[117] Yang J., Li S., Wang Q., Yang D. (2019). Transmembrane Protein 66 Attenuates Neointimal Hyperplasia after Carotid Artery Injury by SOCE Inactivation. Mol. Med. Rep..

[118] Dai F., Zhang Y., Wang Q., Li D., Yang Y., Ma S., Yang D. (2018). Overexpression of SARAF Ameliorates Pressure Overload-Induced Cardiac Hypertrophy Through Suppressing STIM1-Orai1 in Mice. Cell. Physiol. Biochem..

[119] Camargo A., Azuaje F. (2008). Identification of Dilated Cardiomyopathy Signature Genes through Gene Expression and Network Data Integration. Genomics.

[120] Sanlialp A., Schumacher D., Kiper L., Varma E., Riechert E., Ho T.C., Hofmann C., Kmietczyk V., Zimmermann F., Dlugosz S. (2020). Saraf-Dependent Activation of MTORC1 Regulates Cardiac Growth. J. Mol. Cell. Cardiol..

[121] La Russa D., Frisina M., Secondo A., Bagetta G., Amantea D. (2020). Modulation of Cerebral Store-Operated Calcium Entry-Regulatory Factor (SARAF) and Peripheral Orai1 Following Focal Cerebral Ischemia and Preconditioning in Mice. Neuroscience.

[122] Bulla M., Gyimesi G., Kim J.H., Bhardwaj R., Hediger M.A., Frieden M., Demaurex N. (2019). ORAI1 Channel Gating and Selectivity Is Differentially Altered by Natural Mutations in the First or Third Transmembrane Domain. J. Physiol..

[123] Hulot J.-S., Fauconnier J., Ramanujam D., Chaanine A., Aubart F., Sassi Y., Merkle S., Cazorla O., Ouillé A., Dupuis M. (2011). Critical Role for Stromal Interaction Molecule 1 in Cardiac Hypertrophy. Circulation.

[124] Correll R.N., Goonasekera S.A., van Berlo J.H., Burr A.R., Accornero F., Zhang H., Makarewich C.A., York A.J., Sargent M.A., Chen X. (2015). STIM1 Elevation in the Heart Results in Aberrant Ca^2+^ Handling and Cardiomyopathy. J. Mol. Cell Cardiol..

[125] Zhou M.-H., Zheng H., Si H., Jin Y., Peng J.M., He L., Zhou Y., Muñoz-Garay C., Zawieja D.C., Kuo L. (2014). Stromal Interaction Molecule 1 (STIM1) and Orai1 Mediate Histamine-Evoked Calcium Entry and Nuclear Factor of Activated T-Cells (NFAT) Signaling in Human Umbilical Vein Endothelial Cells. J. Biol. Chem..

[126] Gerasimenko J.V., Gerasimenko O.V., Petersen O.H. (2014). The Role of Ca^2+^ in the Pathophysiology of Pancreatitis. J. Physiol..

[127] Eisenhut M., Wallace H. (2011). Ion Channels in Inflammation. Pflug. Arch.

[128] Wen L., Voronina S., Javed M.A., Awais M., Szatmary P., Latawiec D., Chvanov M., Collier D., Huang W., Barrett J. (2015). Inhibitors of ORAI1 Prevent Cytosolic Calcium-Associated Injury of Human Pancreatic Acinar Cells and Acute Pancreatitis in 3 Mouse Models. Gastroenterology.

[129] Gerasimenko J.V., Gryshchenko O., Ferdek P.E., Stapleton E., Hébert T.O.G., Bychkova S., Peng S., Begg M., Gerasimenko O.V., Petersen O.H. (2013). Ca^2+^ Release-Activated Ca^2+^ Channel Blockade as a Potential Tool in Antipancreatitis Therapy. Proc. Natl. Acad Sci. USA.

[130] Kondratska K., Kondratskyi A., Yassine M., Lemonnier L., Lepage G., Morabito A., Skryma R., Prevarskaya N. (2014). Orai1 and STIM1 Mediate SOCE and Contribute to Apoptotic Resistance of Pancreatic Adenocarcinoma. Biochim. Biophys. Acta.

[131] Kim M.S., Hong J.H., Li Q., Shin D.M., Abramowitz J., Birnbaumer L., Muallem S. (2009). Deletion of TRPC3 in Mice Reduces Store-Operated Ca^2+^ Influx and the Severity of Acute Pancreatitis. Gastroenterology.

[132] Crottès D., Lin Y.-H.T., Peters C.J., Gilchrist J.M., Wiita A.P., Jan Y.N., Jan L.Y. (2019). TMEM16A Controls EGF-Induced Calcium Signaling Implicated in Pancreatic Cancer Prognosis. Proc. Natl. Acad. Sci. USA.

[133] Kim J.Y., Zeng W., Kiselyov K., Yuan J.P., Dehoff M.H., Mikoshiba K., Worley P.F., Muallem S. (2006). Homer 1 Mediates Store- and Inositol 1,4,5-Trisphosphate Receptor-Dependent Translocation and Retrieval of TRPC3 to the Plasma Membrane. J. Biol. Chem..

[134] Yuan J.P., Zeng W., Huang G.N., Worley P.F., Muallem S. (2007). STIM1 Heteromultimerizes TRPC Channels to Determine Their Function as Store-Operated Channels. Nat. Cell Biol..

[135] Dong H., Shim K.-N., Li J.M.J., Estrema C., Ornelas T.A., Nguyen F., Liu S., Ramamoorthy S.L., Ho S., Carethers J.M. (2010). Molecular Mechanisms Underlying Ca^2+^-Mediated Motility of Human Pancreatic Duct Cells. Am. J. Physiol. Cell Physiol..

[136] Spinelli A.M., Trebak M. (2016). Orai Channel-Mediated Ca^2+^ Signals in Vascular and Airway Smooth Muscle. Am. J. Physiol. Cell Physiol..

[137] Wang Y.-M., Xu W.-J., Xiang L.-L., Ding M., Zhang J.-J., Lu J.-Y., Xie B.-J., Gao Y.-D. (2021). Store-Operated Calcium Entry-Associated Regulatory Factor Regulates Airway Inflammation and Airway Remodeling in Asthma Mice Models. Am. J. Physiol. Lung Cell Mol. Physiol..

[138] Ng S.W., di Capite J., Singaravelu K., Parekh A.B. (2008). Sustained Activation of the Tyrosine Kinase Syk by Antigen in Mast Cells Requires Local Ca^2+^ Influx through Ca^2+^ Release-Activated Ca^2+^ Channels. J. Biol. Chem..

